# Clinical Practice Guidelines and Consensus Statements in Oncology – An Assessment of Their Methodological Quality

**DOI:** 10.1371/journal.pone.0110469

**Published:** 2014-10-17

**Authors:** Carmel Jacobs, Ian D. Graham, Julie Makarski, Michaël Chassé, Dean Fergusson, Brian Hutton, Mark Clemons

**Affiliations:** 1 Division of Medical Oncology, The Ottawa Hospital Cancer Centre and Department of Medicine, University of Ottawa, Ottawa, Ontario, Canada; 2 Department of Epidemiology and Community Medicine, University of Ottawa, Ottawa, Ontario, Canada; 3 Ottawa Hospital Research Institute and University of Ottawa, Department of Medicine, Ottawa, Ontario, Canada; 4 Independent Consultant, Hamilton, Ontario, Canada; ISPO, Italy

## Abstract

**Background:**

Consensus statements and clinical practice guidelines are widely available for enhancing the care of cancer patients. Despite subtle differences in their definition and purpose, these terms are often used interchangeably. We systematically assessed the methodological quality of consensus statements and clinical practice guidelines published in three commonly read, geographically diverse, cancer-specific journals. Methods Consensus statements and clinical practice guidelines published between January 2005 and September 2013 in Current Oncology, European Journal of Cancer and Journal of Clinical Oncology were evaluated. Each publication was assessed using the Appraisal of Guidelines for Research and Evaluation II (AGREE II) rigour of development and editorial independence domains. For assessment of transparency of document development, 7 additional items were taken from the Institute of Medicine’s standards for practice guidelines and the Journal of Clinical Oncology guidelines for authors of guidance documents.

**Methods:**

Consensus statements and clinical practice guidelines published between January 2005 and September 2013 in Current Oncology, European Journal of Cancer and Journal of Clinical Oncology were evaluated. Each publication was assessed using the Appraisal of Guidelines for Research and Evaluation II (AGREE II) rigour of development and editorial independence domains. For assessment of transparency of document development, 7 additional items were taken from the Institute of Medicine's standards for practice guidelines and the Journal of Clinical Oncology guidelines for authors of guidance documents.

**Findings:**

Thirty-four consensus statements and 67 clinical practice guidelines were evaluated. The rigour of development score for consensus statements over the three journals was 32% lower than that of clinical practice guidelines. The editorial independence score was 15% lower for consensus statements than clinical practice guidelines. One journal scored consistently lower than the others over both domains. No journals adhered to all the items related to the transparency of document development. One journal’s consensus statements endorsed a product made by the sponsoring pharmaceutical company in 64% of cases.

**Conclusion:**

Guidance documents are an essential part of oncology care and should be subjected to a rigorous and validated development process. Consensus statements had lower methodological quality than clinical practice guidelines using AGREE II. At a minimum, journals should ensure that that all consensus statements and clinical practice guidelines adhere to AGREE II criteria. Journals should consider explicitly requiring guidelines to declare pharmaceutical company sponsorship and to identify the sponsor’s product to enhance transparency.

## Introduction

Consensus statements and clinical practice guidelines are widely used in oncology to improve the quality of patient care [1], [2]. While both consensus statements and clinical practice guidelines are intended to provide guidance to clinicians, there are important differences between them. A *clinical practice guideline* (also called a medical guideline or clinical protocol) produces statements that are informed by a systematic review of the evidence and an assessment of the benefits and harms of alternative options [3]. A *consensus statement* is developed by an independent panel of experts, usually multidisciplinary, convened to review the research literature in an evidence-based manner for the purpose of advancing the understanding of an issue, procedure or method [4]. Both documents provide recommendations for optimizing patient care [3].

Although consensus statements address topics in which the evidence base is less extensive compared to clinical practice guidelines, their development should still be methodologically rigorous and transparent [4]. To assist with maintaining methodological rigor, organizations such as Appraisal of Guidelines for Research and Evaluation (AGREE) [5], Institute of Medicine (IOM) [3] and Guidelines International Network (GIN) [6] have developed criteria to ensure objective, scientifically valid, and consistent standards for the development and reporting of high quality guidance documents.

Given their widespread availability and importance for both clinical practice and funding decisions [7], we sought to evaluate the methodological quality of both consensus statements and clinical practice guidelines published in three commonly accessed oncology-specific journals through the domains of rigor of development and editorial independence Information around the transparency of document development was also collected to assess whether or not pharmaceutical company sponsored guidelines were more likely to endorse a product manufactured by the sponsoring company.

## Methods

Three oncology specific journals were searched for consensus statements and clinical practice guidelines published from January 2005–September 2013. Current Oncology (CO), the European Journal of Cancer (EJC) and the Journal of Clinical Oncology (JCO) were chosen as they have editorial offices in different countries and for their perceived prominence in North America and Europe. January 2005 was chosen as the starting date for eligibility, as this was the date by which all three journals had accessible electronic archives. Each journal’s online search tool was used to search for the terms “consensus”, “consensus guideline”, “consensus statement”, “clinical practice guideline”, “practice guideline” or “medical guideline” in the title. Two reviewers (CJ, MC) reviewed each document retrieved to ensure they were consensus statements or practice guidelines, using the IOM criteria “statements that include recommendations intended to optimize patient care” [3].

As our primary focus related to evaluating the methodological quality, we opted to use Domain 3 of the AGREE II tool (Rigour of Development) and Domain 6 (Editorial Independence) to assess the documents. The rigour of development domain consists of 8 items, while the editorial independence domain consists of 2 items (items are shown in Table 1). AGREE II items are scored on a 7-point Likert scale ranging from 1 (strongly disagree) to 7 (strongly agree). Each domain score was calculated as per the AGREE II instructions included in the user’s manual [5]. Domain score = [score obtained – minimum possible score]/[maximum possible score – minimum possible score × 100], giving a percentage score between 0 and 100. As the Standards and Guidelines Evidence (SAGE) directory has used AGREE II to evaluate English language cancer guidelines released since 2003 [8], if a document had been included in the SAGE database, this appraisal was used and a primary assessment of our own was not performed. The SAGE assessment utilises two trained evaluators to assess each document, discrepancies of a certain magnitude are resolved by a third and if required, fourth evaluator [9].

**Table 1:** Items from AGREE II (Domains 3 and 6) and additional items collected to assess Transparency of Document Development.

Criteria collected	Source
**Rigour of development**	
Systematic methods were used to search for evidence.	
The criteria for selecting the evidence are clearly described	
The strengths and limitations of the body of evidence are clearly described.	
The methods for formulating the recommendations are clearly described.	Domain 3 of AGREE II [5]
The health benefits, side effects, and risks have been considered in formulating the recommendations.	
There is an explicit link between the recommendations and the supporting evidence.	
The guideline has been externally reviewed by experts prior to its publication.	
A procedure for updating the guideline is provided.	
**Editorial independence**	
The views of the funding body have not influenced the content of the guideline.	Domain 6 of AGREE II [5]
Competing interests of guideline development group members have been recorded and addressed.	
**Additional items to assess transparency of document development**	
Was a systematic review performed? (yes – systematic review performed anddocumented, no – systematic review not performed or not documented)	IOM [3] JCO [10]
How was the guideline group established? (invited, not disclosed, other),	IOM [3]
Was the group privately funded? (yes, no, not disclosed)	JCO [10].
Was the group multidisciplinary? (yes, no, not disclosed)	JCO [10].
**Consensus sponsor**	
For guidelines where a pharmaceutical product was evaluated was a specific productendorsed in the statement? (yes- name of product, no)	
Name and manufacturer of product endorsed	

IOM  =  Institute of medicine,

JCO  =  Journal of clinical oncology.

As we also wanted to assess issues surrounding the transparency of document development, and specific to whether or not pharmaceutical company sponsorship of the guideline development process was associated with product endorsement, each document was assess using an additional 7 items. These additional items were derived from the IOM standards for trustworthy clinical practice guidelines [3] and the JCO criteria for publishing consensus statements and clinical practice guidelines [10] (Table 1). These items included a statement on “Was a systematic review conducted?” Additional items related to transparency included, “How was the group established?”, “Was the group multidisciplinary?”, “Was the group privately funded?” and “What was the name of the funding body?” In order to assess any relationship between the sponsor of the group and recommendations, for pharmaceutical-related guidelines we also collected data on “Was a specific product endorsed in statement?”, and if so, “Who was the manufacturer of product?”.

Six reviewers appraised the documents, with each document appraised by two independent reviewers (see Acknowledgements). Discrepancy scores between reviewers for AGREE II were calculated using the concordance calculator for the SAGE database calculations [8]. We planned to resolve discrepancies in assessments as per SAGE, by third and if necessary fourth evaluators. For the additional items assessed, any discrepancies between the two reviewers were resolved by consensus.

### Statistical analysis

For the two AGREE II domains of interests, we reported overall means with their 95% confidence intervals for each journal, stratified into separate categories of consensus statement and clinical practice guideline. We also stratified by year of document publication. We used the publication date of the IOM ‘Clinical practice guidelines we can trust’, March 2011 [3], as a time point in which to assess document quality over time. We compared overall differences between journals and between consensus statement or clinical practice guideline using analysis of variance (ANOVA). We also calculated the mean difference in scores between consensus statement and clinical practice guidelines with their corresponding 95% confidence intervals.

For the additional items collected addressing transparency of document development, we calculated the proportion of responses categorized as “Yes”, “No”, and “Not Reported”. We assessed for differences in the journals’ assessments using a chi-square test (or Fisher’s Exact test when dealing with small cell counts in summary contingency tables) at a significance level of 5% while stratifying analyses into categories of consensus statement and clinical practice guideline. Finally, we compared overall items responses according to their consensus statement or clinical practice guideline category.

Agreement between reviewers was assessed by a concordance calculator, determining the number of standard deviations between reviewers, over each domain. A ‘high’ discrepancy score occurred when greater than 2 standard deviations were present between reviewers, ‘medium’ if >1.5 but <2 standard deviations and ‘low’ if <1.5 standard deviations.

## Results

### Identified Literature

The search identified a total of 104 documents for review. Three were excluded as one was a physician survey, one was a review of guidelines, and one was a letter to the editor. Therefore, 34 consensus statements and 67 practice guidelines were retained for assessment. The numbers and types of documents for each journal were; CO-14 consensus statements, 24 clinical practice guidelines, EJC -9 consensus statements, 13 clinical practice guidelines and JCO-11 consensus statements, 30 clinical practice guidelines.

### AGREE II Rigour of development scores

**Figure 1:**
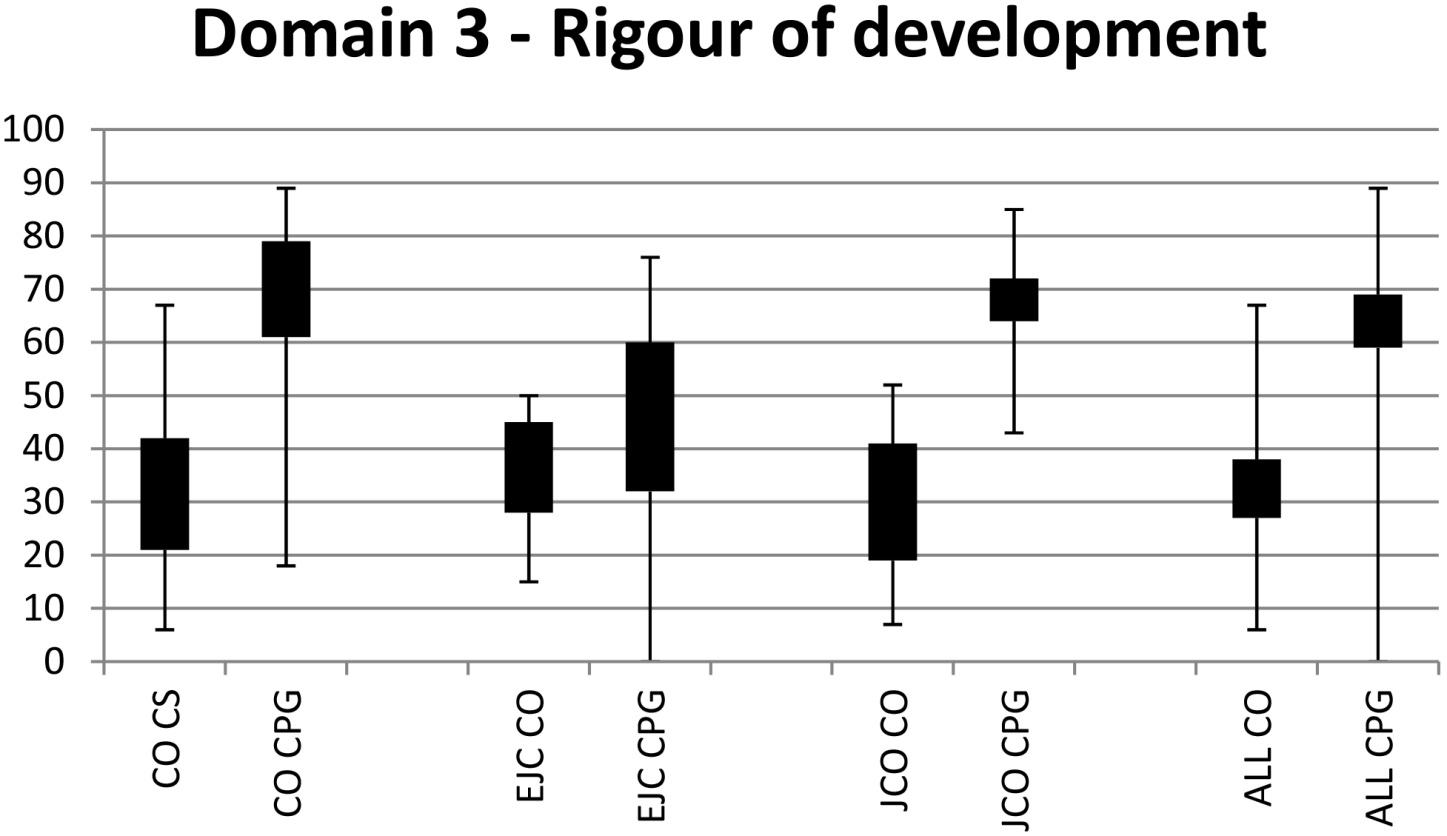
Range and 95% confidence intervals for Rigour of development scores. CO = Current Oncology. EJC = European Journal of Cancer**.** CS = Consensus statements. JCO = Journal of Clinical Oncology**.** CPG = Clinical practice guidelines.

When assessed across all three journals (Figure 1, Table 2), the mean scores for consensus statements were 32% (95% CI 27–38%) and for clinical practice guidelines 64% (95% CI 59–69%). The mean difference between guidelines was 32% (p<0.0001), indicating that clinical practice guidelines were scored significantly higher than consensus statements in terms of rigour of development. Analyses stratified by journal showed that rigour of development scores were significantly lower for consensus statements than clinical practice guidelines for manuscripts published in CO (31% [95% CI 21–40%] consensus statements, 70% [95% CI 61–79%] clinical practice guidelines) and JCO (30% [95% CI 19–41%] consensus statements, 68% [95% CI 64–72%] clinical practice guidelines). There was no significant difference between manuscripts published in EJC (36% [95% CI 28–45%] consensus statements, 46% [95% CI 32–60%] clinical practice guidelines). When comparing each journal with the others, all three had similar scores for consensus statements; however EJC clinical practice guidelines scored lower than Current Oncology (EJC 46% [95% CI 32–60%], CO 70% [95% CI 61–79%]) and JCO (68% [95% CI 64–72%]). Discrepancy levels between the reviewers were low with the exception of one consensus statement published in the Journal of Clinical Oncology [11] which had a high discrepancy score.

**Table 2:** AGREE II: Rigour of development and Editorial Independence.

	CO	EJC	JCO	Overall	p-value, difference between means
**AGREE II: Rigour of development (Domain 3)**					
**Consensus Statement (n = 34)**					
**Mean (95% Confidence Interval)**	31 (21, 42)	36 (28, 45)	30 (19, 41)	32 (27, 38)	0.6400
**Clinical Practice Guideline (n = 67)**					
**Mean (95% Confidence Interval)**	70 (61, 79)	46 (32, 60)	68 (64, 72)	64 (59, 69)	0.0006
**Mean difference Consensus Statement vs Clinical Practice Guideline**					32 (24, 40)
**Overall p-value Consensus Statement vs Clinical Practice Guideline**					<0.0001
**AGREE II: Editorial Independence (Domain 6)**					
**Consensus Statement (n = 34)**					
**Mean (95% Confidence Interval)**	50 (38, 62)	44 (34, 54)	63 (56, 70)	53 (47, 59)	0.0305
**Clinical Practice Guideline (n = 67)**					
**Mean (95% Confidence Interval)**	75 (63, 86)	59 (52, 67)	66 (61, 70)	68 (63, 73)	0.0564
**Mean difference Consensus Statement vs Clinical Practice Guideline**					15 (7, 23)
**Overall p-value Consensus Statement vs Clinical Practice Guideline**					0.0003

### AGREE II Editorial independence scores

**Figure 2:**
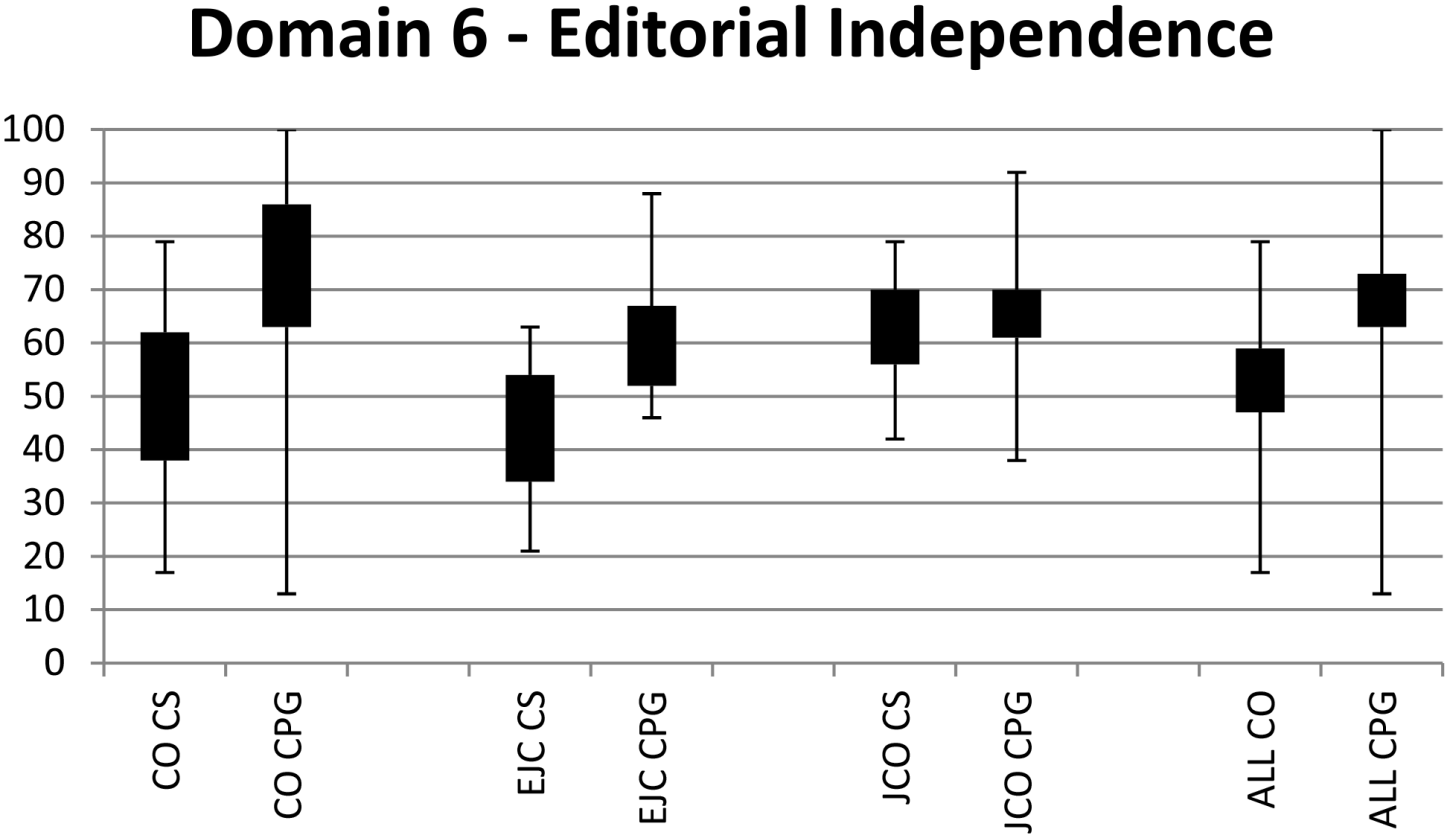
Range and 95% confidence intervals for Editorial independence scores. CO = Current Oncology. EJC = European Journal of Cancer. CS = Consensus statements. JCO = Journal of Clinical Oncology. CPG = Clinical practice guidelines.

When assessed across all three journals (Figure 2, Table 2), the mean score for consensus statements was 53% (95% CI 47–59%) and for clinical practice guidelines was 68% (95% CI 63–73%). The mean difference between consensus statement and clinical practice guideline scores was 15% (p  = 0.0003), indicating that clinical practice guidelines were scored significantly higher than consensus statements with respect to editorial independence. Editorial independence scores were significantly lower for consensus statements than clinical practice guidelines in documents published in CO (50% [95% CI 38–62%] consensus statements, 75% [95% CI 63–86%] clinical practice guidelines). This difference seen to a lesser extent in EJC (44% [95% CI 34–54%] consensus statements, 59% [95% CI 52–67%] clinical practice guidelines) and no difference was seen in JCO (63% [95% CI 56–70%] consensus statements, 66% [95% CI 61–70%] clinical practice guidelines). EJC (44% [95% CI 35–54%]) scored lower than JCO (63% [95% CI 56–70%]) on consensus statements, but similarly to CO. No journal appeared to perform better or worse than the other journals with regard to clinical practice guidelines. Discrepancy levels between the reviewers were low for all documents.

### Additional transparency of document development item scores

Consensus statements infrequently referenced or conducted a systematic review on the topic of the guideline (6/34 = 18%), a step which was much more common with clinical practice guidelines (56/67 = 83%) (p = 0.018) (Table 3). The largest discrepancy was seen in JCO where 0/11 (0%) of consensus statements documented a systematic review compared to 28/30 (93%) of clinical practice guidelines. Neither consensus statements (50%) nor clinical practice guidelines (34%) consistently declared how their development group was established. Consensus statements were more likely than clinical practice guidelines to state that participants were “invited” (12/34 = 35% vs 14/67 = 21%; p = 0.01). Guideline groups were multidisciplinary in 21 out of 34 (62%) consensus statements and 50 out of 67 (75%) clinical practice guidelines groups. Group member roles were not declared in 35% (12/34) of the consensus statements nor in 25% (17/67) of clinical practice guidelines (p = 0.19).

**Table 3:** Additional items addressing Transparency of Document Development.

	CO n (%)	EJC n (%)	JCO n (%)	Overall n (%)	p-value
**Systematic review performed**					
**Consensus Statement yes (n = 34)**	3 (21)	3 (33)	0	6 (18)	0.1350
**Clinical Practice Guideline yes (n = 67)**	21 (88)	7 (54)	28 (93)	56 (84)	0.0082
**Overall Consensus Statement vs Clinical Practice Guideline difference**					<0.0001
**How groups were established**					
**Consensus Statement (n = 34)**					
**Invited**	6 (43)	5 (56)	1 (9)	12 (35)	0.1440
**Not reported**	6 (43)	4 (44)	7 (64)	17 (50)	
**Other**	2 (14)	0	3 (27)	5 (15)	
**Clinical Practice Guideline (n = 67)**					
**Invited**	4 (17)	5 (39)	5 (17)	14 (21)	0.0378
**Not reported**	7 (29)	7 (54)	9 (30)	23 (34)	
**Other**	13 (54)	1 (8)	16 (53)	30 (45)	
**Overall Consensus Statement vs Clinical Practice Guideline difference**					0.0106
**Multidisciplinary**					
**Consensus Statement (n = 34)**					
**Yes**	8 (57)	7 (78)	6 (55)	21 (62)	0.7182
**No**	1 (7)	0	0	1 (3)	
**Not reported**	5 (36)	2 (22)	5 (46)	12 (35)	
**Clinical Practice Guideline (n = 67)**					
**Yes**	19 (79)	8 (62)	23 (77)	50 (75)	0.4716
**No**	0	0	0	0	
**Not reported**	5 (21)	5 (39)	7 (23)	17 (25)	
**Overall Consensus Statement vs Clinical Practice Guideline difference**					0.1857
**Privately funded meeting**					
**Consensus Statement (n = 34)**					
**Yes**	9 (64)	2 (22)	0	11 (32)	<0.0001
**No**	1 (7)	0	0	1 (3)	
**Not reported**	4 (29)	7 (78)	11 (100)	22 (65)	
**Clinical Practice Guideline (n = 67)**					
**Yes**	1 (4)	5 (39)	0	6 (9)	<0.0001
**No**	15 (63)	0	16 (53)	31 (47)	
**Not reported**	8 (33)	8 (62)	14 (47)	30 (45)	
**Overall Consensus Statement vs Clinical Practice Guideline difference**					<0.0001
**Consensus sponsors’ product endorsed**					
**Consensus Statement (n = 34)**					
**Yes**	9 (64)	1 (11)	0	10 (24.4)	<0.0001
**Clinical Practice Guideline (n = 67)**					
**Yes**	1 (4)	1 (8)	0	2 (3)	0.3012
**Overall Consensus Statement vs Clinical Practice Guideline difference**					<0.0001

While consensus statements were more likely to declare private funding (11/34 = 32%) than clinical practice guidelines (6/67 = 9%) (p<0.0001), many documents did not declare their source of funding (22/34 = 65% of consensus statements versus 31/67 = 46% of clinical practice guidelines). If a source of funding was declared, the funding body was recorded (Table 3).

With respect to whether or not a document endorsed a product made by the sponsoring company (Table 3), this occurred less frequently in clinical practice guidelines (2/67 = 3%) than in consensus statements (10/34 = 29%) (p<0.0001). In CO, consensus statements endorsed the product of the sponsoring company in 9/14 (64%) of cases. All of these documents declared financial support from the sponsoring company, but none explicitly declared the link between the sponsoring company and the product endorsed. Four percent of clinical practice guidelines published in CO endorsed the sponsor’s product. This trend was seen to a lesser extent in EJC with 11% of consensus statements endorsing sponsors products and 8% of clinical practice guidelines. No document published by JCO documented a relationship between pharmaceutical company funding and product endorsement in the guideline.

### Have consensus statements and clinical practice guidelines improved over time?

When assessed chronologically, there is no association with document quality over time, using the date of publication of the IOM ‘Clinical practice guidelines we can trust’, March 2011 as a reference point (Tables 4,5 and 6). There may be a trend of declining pharmaceutical sponsorship of documents in recent years.

**Table 4:** Current Oncology Consensus Statements and Clinical Practice Guidelines.

Current Oncology												
*Consensus Statements*												
Paper	Year published			Pharma sponsored		AGREE Domain 3 (%)		AGREE Domain 6 (%)				Sponsors product endorsed
Recommendations of the Canadian Consensus Group onthe Management of Chronic Myeloid Leukemia [12]	2006			✓		50		50				✓
Updated recommendations from the Canadian Nationalconsensus meeting on HER2/neu testing in breast cancer[13]	2007			✓		7		17				✓
Colorectal Cancer Association of Canada ConsensusMeeting on Practice Guidelines - Raising the Standard ofCare in Canada for Early Stage Rectal Cancer [14]	2009			✓		22		64				✓
The role of the Epidermal Growth Factor Receptor TyrosineKinase Inhibitors as Therapy for Advanced, Metastatic andRecurrent Non-Small Cell Lung Cancer: A Canadian NationalConsensus Statement [15]	2009			✓		67		46				✓
Consensus recommendations for the use of anti-EGFRtherapies in metastatic colorectal cancer [16]	2010					6		54				
Eastern Canadian Colorectal Cancer ConsensusConference: setting the limits of resectable disease [17]	2010			✓		32		21				✓
Consensus recommendations for the diagnosis andmanagement of well-differentiated gastroenterohepaticneuroendocrine tumours: a revised statement from aCanadian national expert group [18]	2010			✓		23		79				✓
Diagnosis and management of hepatocellular carcinoma:results of a consensus meeting of The Ottawa Hospital Cancer Centre [19]	2010			✓		33		25				✓
**Consensus Statements pre IOM 2011 (n = 8), Mean (95% Confidence Interval)**	**30 (16, 44)**			**45 (29, 60)**								
Multidisciplinary Canadian consensus recommendations forthe management and treatment of hepatocellular carcinoma [20]	2011			✓		56		75			✓	
Canadian Expert Group consensus recommendations: KRAStesting in colorectal cancer [21]	2011					19		58				
Report from the 13th Annual Western CanadianGastrointestinal Cancer Consensus Conference [22]	2012					43		46				
Consensus recommendations for cancer rehabilitation:research and education priorities [23]	2013					14		79				
Endocrine therapy for postmenopausal women withhormone receptor–positive her2–negative advancedbreast cancer after progression or recurrence on nonsteroidal aromatase inhibitor therapy: a Canadian consensus statement [24]	2013			✓		40		63			✓	
Eastern Canadian Colorectal Cancer ConsensusConference: standards of care for the treatment of patients with rectal, pancreatic, andgastrointestinal stromaltumours and pancreatic neuroendocrine tumours [25]	2013						29		33			
**Consensus Statements post IOM 2011 (n = 6), Mean (95% Confidence Interval)**							**34 (21, 46)**		**59 (45, 73)**			
***Clinical Practice Guidelines***												
Bortezomib in Multiple Myeloma and Lymphoma: Asystematic review and clinical practice guideline [26]	2006				82				88			
Guidelines for the diagnosis and management of carcinoidtumours, part 1: the gastrointestinal tract. A statement from a Canadian National Carcinoid Expert Group [27]	2006				20				13			
The role of oxaliplatin combined with 5-fluorouracil andfolinic acid in the first- and second-line treatment of advanced colorectal cancer: a systematic review and clinical practice guideline [28]	2006				74				21			
Canadian Recommendations for the Treatment ofGlioblastoma Multiforme [29]	2007			✓	19				17		✓	
Melanoma Disease Site Group of Cancer Care Ontario’sProgram in Evidence-Based Care Mot. Temozolomide for the Treatment of MetastaticMelanoma: A PracticeGuideline [30]	2007				80				92			
Ifosfamide-based combination chemotherapy in advancedsoft tissue sarcoma: a systematic review and clinical practice guideline [31]	2007				61				96			
Management of Single Brain Metastases: A PracticeGuideline [32]	2007				79				92			
Alemtuzumab in Chronic Lymphocytic Leukemia: ASystematic Review and Clinical Practice Guideline [33]	2007				81				100			
Single-Agent Interleukin-2 in the Treatment of MetastaticMelanoma: A Clinical Practice Guideline [34]	2007				78				100			
Biochemotherapy for the Treatment of MetastaticMalignant Melanoma: A Clinical Practice Guideline [35]	2008				43				75			
Dose-intensive Chemotherapy with Growth Factor or Autologous Bone Marrow/Stem Cell Transplant Support inFirst-line Treatment of Advanced or MetastaticAdult Soft Tissue Sarcoma – A Clinical PracticeGuideline [36]	2008				89				100			
Epidermal growth factor receptor targeted therapy in stageIII and IV head and neck cancer [37]	2010				85				79			
Follow-up for women after treatment for cervical cancer[38]	2010				78				79			
**Clinical Practice Guidelines pre IOM 2011 (n = 13) Mean (95% Confidence Interval)**					**67 (54, 80)**				**73 (55, 91)**			
Canadian College of Medical Geneticists guidelines for theindications, analysis, and reporting of cancer specimens [39]		2011			18				46			
Systemic therapy for advanced gastric cancer: a clinicalpractice guideline [40]		2011			84				83			
Survivorship services for adult cancer populations: a pan-Canadian guideline [41]		2011			84				96			
Invasive mediastinal staging of non-small-cell lung cancer: aclinical practice guideline [42]		2011			75				83			
Management of a suspicious adnexal mass: a clinicalpractice guideline [43]		2012			80				63			
Lenalidomide in multiple myeloma – a practice guideline[44]		2013			75				83			
Chemotherapy (gemcitabine, docetaxel plus gemcitabine,doxorubicin, or trabectedin) in inoperable, locallyadvanced, recurrent, or metastatic uterineleiomyosarcoma: a clinical practice guideline [45]		2013			82				88			
Role of endolaryngeal surgery (with or without laser)compared with radiotherapy in the managementof early (T1) glottic cancer: a clinical practiceguideline [46]		2013			74				79			
Surgical margins and handling of soft-tissue sarcoma inextremities: a clinical practice guideline [47]		2013			77				100			
Liver resection for colorectal cancer metastases [48]		2013			80				83			
A pan-Canadian practice guideline and algorithm:screening, assessment, and supportive care ofadults with cancer-related fatigue [49]		2013			75				38			
**Clinical Practice Guidelines post IOM 2011 (n = 11) Mean (95% Confidence Interval)**					**73 (62, 84)**				**77 (65, 88)**			

**Table 5:** European Journal of Cancer Consensus Statements and Clinical Practice Guidelines.

European Journal of Cancer					
*Consensus Statements*					
Paper	Year published	Pharma sponsored	AGREE Domain 3	AGREE Domain 6	Sponsors product endorsed
EORTC consensus recommendations for the treatment ofmycosis fungoides/Sézary syndrome [50]	2006		43	50	
Towards a pan-European consensus on the treatment ofpatients with colorectal liver metastases. [51]	2006		41	38	
Consensus conference: Implementing treatmentrecommendations on yttrium-90 immunotherapy inclinical practice – Report of a European workshop [52]	2008	✓	27	42	✓
Diagnosis and treatment of melanoma: Europeanconsensus-based interdisciplinary guideline [53]	2010		39	38	
Breast cancer in pregnancy: Recommendations of aninternational consensus meeting. [54]	2010		49	21	
**Consensus Statements pre IOM 2011 (n = 8), Mean (95% Confidence Interval)**			**40 (33, 47)**	**38 (29, 47)**	
Consensus on Lung Cancer, new clinical recommendationsand current status of biomarker assessment – First-line therapy. [55]	2011		15	38	
Highlights of the EORTC St. Gallen International ExpertConsensus on the primary therapy of gastric,gastroesophageal and oesophageal cancer – Differentialtreatment strategies for subtypes of earlygastroesophageal cancer. [56]	2012	✓	28	63	
Diagnosis and treatment of melanoma. Europeanconsensus-based interdisciplinary guideline – Update 2012 [57]	2012		35	63	
German, Austrian and Swiss consensus conference on thediagnosis and local treatment of the axilla in breast cancer [58]	2013		50	46	
**Consensus Statements post IOM 2011 (n = 4), Mean (95% Confidence Interval)**			**32 (18, 46)**	**53 (40, 65)**	
***Clinical Practice Guidelines***					
Guidelines for surgical treatment of hepatoblastoma in themodern era–recommendations from the Childhood Liver Tumour Strategy Group of the International Society ofPaediatric Oncology (SIOPEL) [59]	2005		21	46	
Malignant ascites: systematic review and guideline for treatment. [60]	2006		63	46	
EORTC guidelines for the use of granulocyte-colonystimulating factor to reduce the incidence of chemotherapy-induced febrile neutropenia in adultpatients with lymphomas and solid tumours. [61]	2006	✓	72	50	
EORTC guidelines for the use of erythropoietic proteins inanaemic patients with cancer: 2006 update [62]	2007	✓	60	88	
Tumour markers in colorectal cancer: European Group onTumour Markers (EGTM) guidelines for clinical use [63]	2007		38	64	
Guidelines on the standards for the training of specialisedhealth professionals dealing with breast cancer [64]	2007	✓	0	46	
Guidelines for the assessment of oral mucositis in adultchemotherapy, radiotherapy and haematopoietic stem cell transplant patients. European journal of cancer [65]	2008		69	58	
Diagnosis and antimicrobial therapy of lung infiltrates infebrile neutropenic patients: Guidelines of the infectiousdiseases working party of the German Society of Haematology and Oncology. [66]	2009		18	58	
The development of evidence-based guidelines on mouthcare for children, teenagers and young adults treated for cancer [67]	2010		76	63	
2010 update of EORTC guidelines for the use ofgranulocyte-colony stimulating factor to reduce the incidence of chemotherapy-induced febrile neutropeniain adult patients with lymphoproliferative disorders and solid tumours [68]	2011	✓	63	50	✓
The development of evidence-based Europeanguidelines on the management of depression inpalliative cancer care [69]	2011		41	71	
**Clinical Practice Guidelines pre IOM 2011 (n = 11) Mean (95% Confidence Interval)**			**47 (32, 62)**	**58 (51, 66)**	
Paediatric intestinal cancer and polyposis due to bi-allelic PMS2 mutations: case series, review and follow-up guidelines. European journal of cancer[70]	2011		28	75	
EASL-EORTC clinical practice guidelines:management of hepatocellular carcinoma [71]	2012		49	58	
**Clinical Practice Guidelines post IOM 2011 (n = 2) Mean (95% Confidence Interval)**			**39 (18, 59)**	**67 (50, 83)**	

**Table 6:** Journal of Clinical Oncology Consensus Statements and Clinical Practice Guidelines.

Journal of Clinical Oncology					
*Consensus Statements*					
Paper	Year published	Pharma sponsored	AGREE Domain 3	AGREE Domain 6	Sponsors product endorsed
Use of Positron Emission Tomography for ResponseAssessment of Lymphoma: Consensus of the ImagingSubcommittee of International Harmonization Project inLymphoma. [72]	2007		49	63	
Definition, diagnosis, and management of intravascular large B-cell lymphoma: proposals and perspectives from aninternational consensus meeting. [73]	2007		9	67	
Consensus Report of the National Cancer Institute ClinicalTrials Planning Meeting on Pancreas Cancer Treatment [74]	2009		7	67	
Venous Thromboembolism Prophylaxis and Treatment inCancer: A Consensus Statement of Major Guidelines Panelsand Call to Action [11]	2009		52	71	
Definition, Prognostic Factors, Treatment, and ResponseCriteria of Adult T-Cell Leukemia-Lymphoma: A Proposal Froman International Consensus Meeting. [75]	2009		38	67	
International Myeloma Working Group Consensus StatementRegarding the Current Status of Allogeneic Stem-CellTransplantation for Multiple Myeloma [76]	2010		23	46	
Renal Impairment in Patients With Multiple Myeloma: AConsensus Statement on Behalf of the International Myeloma Working Group [77]	2010		29	42	
Hepatocellular Carcinoma: Consensus Recommendations ofthe National Cancer Institute Clinical Trials Planning Meeting [78]	2010		35	67	
Future Directions in the Treatment of NeuroendocrineTumors: Consensus Report of the National Cancer Institute Neuroendocrine Tumor Clinical Trials Planning Meeting. [79]	2011		30	67	
**Consensus Statements pre IOM 2011 (n = 9), Mean (95% Confidence Interval)**			**30 (20,40)**	**62 (55,69)**	
Clinical End Points and Response Criteria in Mycosis Fungoidesand Sézary Syndrome: A Consensus Statement of the International Society for Cutaneous Lymphomas, the UnitedStates Cutaneous Lymphoma Consortium, and the Cutaneous Lymphoma Task Force of the EuropeanOrganisation for Research and Treatment of Cancer. [80]	2011		8	63	
Platinum-Induced Ototoxicity in Children: A Consensus Review on Mechanisms, Predisposition, and Protection, Including a New International Society of Pediatric Oncology Boston Ototoxicity Scale. [81]	2012		45	79	
**Consensus Statements post IOM 2011 (n = 29), Mean (95% Confidence Interval)**			**27 (0,63)**	**71 (55,87)**	
***Clinical Practice Guidelines***					
American Society of Clinical Oncology GuidelineRecommendations for Sentinel Lymph Node Biopsy inEarly-Stage Breast Cancer. [82]	2005		75	73	
Colorectal Cancer Surveillance: 2005 Update of anAmerican Society of Clinical Oncology Practice Guideline [83]	2005		65	79	
American Society of Clinical Oncology/College of American Pathologists Guideline Recommendations for HumanEpidermal Growth Factor Receptor 2 Testing in BreastCancer. [84]	2006		82	65	
2006 Update of Recommendations for the Use of WhiteBlood Cell Growth Factors: An Evidence-Based Clinical Practice Guideline. [85]	2006		57	77	
American Society of Clinical Oncology Clinical PracticeGuideline for the Use of Larynx-Preservation Strategies in the Treatment of Laryngeal Cancer. [86]	2006		65	54	
American Society of Clinical Oncology Guideline forAntiemetics in Oncology: Update 2006. [87]	2006		65	64	
American Society of Clinical Oncology Guideline:Recommendations for Venous Thromboembolism Prophylaxis and Treatment in Patients With Cancer. [88]	2007		81	63	
Cancer Care Ontario and American Society of ClinicalOncology Adjuvant Chemotherapy and Adjuvant RadiationTherapy for Stages I-IIIA Resectable Non–Small-Cell Lung Cancer Guideline [89].	2007		79	88	
American Society of Clinical Oncology Endorsement of theCancer Care Ontario Practice Guideline on Nonhormonal Therapy for Men With Metastatic Hormone-Refractory (castration-resistant) Prostate Cancer. [90]	2007		69	92	
American Society of Clinical Oncology 2007 ClinicalPractice Guideline Update on the Role of Bisphosphonatesin Multiple Myeloma. [91]	2007		60	75	
Initial Hormonal Management of Androgen-SensitiveMetastatic, Recurrent, or Progressive Prostate Cancer:2007 Update of an American Society of Clinical Oncology Practice Guideline. [92]	2007		75	38	
American Society of Clinical Oncology 2008 ClinicalPractice Guideline Update: Use of Chemotherapy andRadiation Therapy Protectants. [93]	2009		70	58	
Use of 5-alpha-reductase inhibitors for prostate cancerchemoprevention: American Society of Clinical Oncology/American Urological Association 2008 ClinicalPractice Guideline. [94]	2009		76	71	
American Society of Clinical Oncology Clinical Practice Guideline Update on Chemotherapy for Stage IV Non–Small-Cell Lung Cancer [95].	2009		71	71	
American Society of Clinical Oncology Clinical PracticeGuideline Update on the Use of Pharmacologic Interventions Including Tamoxifen, Raloxifene, andAromatase Inhibition for Breast Cancer Risk Reduction [96].	2009		74	63	
American Society of Clinical Oncology/American Society ofHematology Clinical Practice Guideline Update on the Use of Epoetin and Darbepoetin in Adult Patients With Cancer [97].	2010		57	54	
American Society of Clinical Oncology Clinical PracticeGuideline: Update on Adjuvant Endocrine Therapy for Women With Hormone Receptor–Positive Breast Cancer[98].	2010		54	54	
American Society of Clinical Oncology Clinical PracticeGuideline on Uses of Serum Tumor Markers in Adult Males With Germ Cell Tumors [99].	2010		58	63	
American Society of Clinical Oncology/College of AmericanPathologists Guideline Recommendations forImmunohistochemical Testing of Estrogen andProgesterone Receptors in Breast Cancer [100].	2010		65	47	
**Clinical Practice Guidelines pre IOM 2011 (n = 19) Mean (95% Confidence Interval)**			**69 (64,72)**	**66 (60,72)**	
Antiemetics: American Society of Clinical Oncology ClinicalPractice Guideline Update [101].	2011		72	67	
American Society of Clinical Oncology Endorsement of theCancer Care Ontario Practice Guideline on Adjuvant Ovarian Ablation in the Treatment of PremenopausalWomen With Early-Stage Invasive Breast Cancer [102].	2011		85	79	
2011 Focused Update of 2009 American Society of ClinicaOncology Clinical Practice Guideline Update on Chemotherapy for Stage IV Non–Small-Cell Lung Cancer[103].	2011		43	58	
American Society of Clinical Oncology Clinical PracticeGuideline Update on the Use of Chemotherapy Sensitivity and Resistance Assays [104].	2011		54	58	
Sentinel Lymph Node Biopsy for Melanoma: AmericanSociety of Clinical Oncology and Society of SurgicalOncology Joint Clinical Practice Guideline [105].	2012		63	75	
Appropriate Chemotherapy Dosing for Obese AdultPatients With Cancer: American Society of Clinical Oncology Clinical Practice Guideline [106].	2012		67	54	
Use of Pharmacologic Interventions for Breast Cancer RiskReduction: American Society of Clinical Oncology ClinicalPractice Guideline [107].	2013		82	63	
Fertility Preservation for Patients With Cancer: AmericanSociety of Clinical Oncology Clinical Practice GuidelineUpdate [108].	2013		65	71	
Central Venous Catheter Care for the Patient With Cancer:American Society of Clinical Oncology Clinical PracticeGuideline [109].	2013		80	58	
Breast Cancer Follow-Up and Management After PrimaryTreatment: American Society of Clinical Oncology Clinical Practice Guideline Update [110].	2013		48	71	
Antimicrobial Prophylaxis and Outpatient Management ofFever and Neutropenia in Adults Treated for Malignancy: American Society of Clinical Oncology Clinical Practice Guideline [111].	2013		81	71	
**Clinical Practice Guidelines post IOM 2011 (n = 11) Mean (95% Confidence Interval)**			**67 (59, 76)**	**66 (61, 71)**	

## Discussion

As the terms consensus statement and clinical practice guidelines are often used interchangeably and both are used to improve clinical care, their methodological rigour and transparency of development is essential. Here we report the results of a review of the methodological quality of consensus statements and clinical practice guidelines in a limited sample of the oncology literature. While others have published on quality assessment of clinical guidelines in oncology using either the AGREE or AGREE II tool [112]–[117], to our knowledge this is the first such comprehensive review of both consensus statement and practice guidelines in oncology.

As literature assessing the quality of consensus statements is limited [118], we used tools developed for clinical practice guidelines and collected additional information that would help assess the transparency of guideline development. AGREE II is a validated appraisal tool for assessing the methodological development quality and reporting of practice guidelines; it does not assess the actual content of clinical recommendations [5]. AGREE II assesses how well a guideline performs on each of the 6 domains (scope and purpose, stakeholder involvement, rigour of development, clarity of presentation, applicability and editorial independence). We felt the rigour of development (an assessment of the evidentiary base and methods used to formulate recommendations) and editorial independence (an assessment of bias and competing interests influencing recommendation formulation [5]) were the most appropriate for our evaluation.

For both the rigour of development and editorial independence domains, consensus statements scored consistently less well than did practice guidelines. In the only publication we found evaluating practice guidelines in comparison to consensus statements, although not specific to oncology [118], similar differences were seen, with consensus statements scoring significantly lower than clinical practice guidelines across 4 of the 6 AGREE II domains (stakeholder involvement, rigour of development, clarity, and presentation and applicability). We could show no improvement in document quality over time.

Performing a systematic review is an essential element of guideline development [119]. Both IOM [120] and JCO [10] state that “clinical practice guideline developers should use systematic reviews” and that “guidelines/recommendations should be driven by a high level of evidence” respectively. We felt it was necessary to specifically ask ‘was a systematic review performed?’ We asked this question even though AGREE II domain 3.1 assess if ‘systematic methods were used to search for evidence’ (scored on a continuum of whether a guideline reports what databases were searched, the search terms used, the search time periods and the inclusion of a full search strategy). In the current study systematic reviews were performed more frequently by clinical practice guidelines than consensus statements across all three journals. With respect to the processes by which a clinical practice guideline group was established and the role of individual members, this was inconsistently reported. There were however significant differences between these items in consensus statements and clinical practice guidelines.

Of particular interest was the role of the funding body for the development of the guidance document. While no information can be gleaned for whether this association is real or implied, several observations can be made. Overall, consensus statements and clinical practice guidelines published in the three journals studied either did not declare or were not explicit about the funding source for the document (funding source not declared in 65% consensus statements, 45% clinical practice guidelines). For documents with topics related to pharmaceutical products, when the document was sponsored by a pharmaceutical company, documents endorsed the sponsor’s product in both consensus statements (29%) and to a lesser degree in clinical practice guidelines (3%). However, in the CO journal, 64% of consensus statements published endorsed the sponsors product, whereas only 4% of clinical practice guidelines endorsed the sponsors product. Further, this association was not reported in the conflict of interest statement. This absence of reporting contravenes standards published by medical societies [121], [122] and could question the integrity and quality of published guidance documents [123], [124].

We acknowledge a number of study limitations. Although we feel that consensus statements should be subjected to the same rigorous criteria for their development as practice guidelines, the AGREE II tool has not been validated for evaluation of consensus statements [5], [118]. The additional items we included for assessment from the IOM guideline standards and JCO authorship guidance on consensus statements and clinical practice guidelines also have not been validated. Consensus statements and clinical practice guidelines analyzed here may not be representative of all oncology consensus statements and clinical practice guidelines released between January 2005 and September 2013, nor representative of all oncology journals. A brief PubMed search suggests over 900 oncology guidance documents were published in peer-reviewed journals over the same time period, translating to a sample of 11% of these documents. Finally, we chose only three journals from which to sample. Our rational for selecting them was that they commonly publish both consensus statements and clinical practice guidelines, are prominent journals in their locale of origin and are geographically diverse. We appreciate that these journals may not be representative of all oncology journals.

## Conclusions

While consensus statements and clinical practice guidelines are developed with slightly different approaches and methods, both are used to inform clinical and policy decisions. As such both documents should be developed using equally rigorous and transparent methods and subjected to high quality standards. Here we have shown that consensus statements score lower than clinical practice guidelines for scores of rigour of development and editorial independence. Consensus statements are also less likely to include a systematic review of the literature and were more likely to be sponsored by a pharmaceutical company and to endorse a specific pharmaceutical product. Unfortunately transparency of document development was generally poor in both types of documents and there was infrequent documentation of sources of funding, how guideline groups were established and who comprised their guideline development groups.

Given the important role of guidance we feel that both consensus statements and clinical practice guidelines should be subject to the same rigorous and high quality development criteria. We suggest that journals encourage authors of guidance documents to use the AGREE II and IOM criteria when developing their documents and require journal reviewers to use these same criteria when undertaking their peer-review of these documents. While there are quality differences between each of the journals sampled in our study, this was most pronounced around the issues of private funding and product endorsement. Readers of guidance documents published within these journals should be made aware of the presence of private funding and sponsorship should be made transparent through their reporting so that readers can acknowledge such conflicts and potential bias.

## References

[1] Woolf SH, Grol R, Hutchinson A, Eccles M, Grimshaw J (1999) Potential benefits, limitations, and harms of clinical guidelines. BMJ 318: 527–530. 10024268 10.1136/bmj.318.7182.527 PMC1114973

[2] Grimshaw JM, Russell IT (1993) Effect of clinical guidelines on medical practice: a systematic review of rigorous evaluations. Lancet 342: 1317–1322. 7901634 10.1016/0140-6736(93)92244-n

[3] Graham RMM, Miller Wolman D, Greenfield S, Steinberg E, Editors, Committee on Standards for Developing Trustworthy Clinical Practice Guidelines Clinical Practice Guidelines We Can Trust.: Institute of Medicine.

[4] (2009) Mosby's Medical Dictionary. 8th Edition ed: Elsevier.

[5] Brouwers MC, Kho ME, Browman GP, Burgers JS, Cluzeau F, et al (2010) AGREE II: advancing guideline development, reporting and evaluation in health care. Cmaj 182: E839–842. 20603348 10.1503/cmaj.090449 PMC3001530

[6] Guidelines International Network. Available: http://www.g-i-n.net/. Accessed 26 May 2014.

[7] Canadian partnership against cancer and pCODR. (2013) How cancer drug funding decisions are made Available: http://www.pcodr.ca/idc/groups/pcodr/documents/pcodrdocument/pcodr-funding-tutorial.pdf. Accessed 28 April 2014.

[8] Brouwers (2010) Standards and Guidelines Evidence (SAGE) Directory of Cancer Guidelines. Available: http://www.cancerview.ca/cv/portal/Home/TreatmentAndSupport/TSProfessionals/ClinicalGuidelines/GRCMain/GRCSAGE?_afrLoop=176960541903000&lang=en&_afrWindowMode=0&_adf.ctrl-state=n8sm6f4dx_85. Accessed 28 September 2013.

[9] Brouwers MC, Rawski E, Spithoff K, Oliver TK (2011) Inventory of Cancer Guidelines: a tool to advance the guideline enterprise and improve the uptake of evidence. Expert Rev Pharmacoecon Outcomes Res 11: 151–161. 21476817 10.1586/erp.11.11

[10] Oncology JoC Information for Contributors.

[11] Khorana AA, Streiff MB, Farge D, Mandala M, Debourdeau P, et al (2009) Venous Thromboembolism Prophylaxis and Treatment in Cancer: A Consensus Statement of Major Guidelines Panels and Call to Action. Journal of Clinical Oncology 27: 4919–4926. 19720907 10.1200/JCO.2009.22.3214 PMC2799060

[12] Laneuville P, Barnett MJ, Belanger R, Couban S, Forrest DL, et al (2006) Recommendations of the canadian consensus group on the management of chronic myeloid leukemia. Curr Oncol 13: 201–221. 22792021 10.3747/co.v13i6.124 PMC3394607

[13] Hanna W, O'Malley F P, Barnes P, Berendt R, Gaboury L, et al (2007) Updated recommendations from the Canadian National Consensus Meeting on HER2/neu testing in breast cancer. Curr Oncol 14: 149–153. 17710207 10.3747/co.2007.131 PMC1948868

[14] Colorectal Cancer Association of Canada consensus meeting: raising the standards of care for early-stage rectal cancer (2009) Curr Oncol. 16: 50–56. 10.3747/co.v16i6.505 PMC2794673 20016746

[15] Ellis PM, Morzycki W, Melosky B, Butts C, Hirsh V, et al (2009) The role of the epidermal growth factor receptor tyrosine kinase inhibitors as therapy for advanced, metastatic, and recurrent non-small-cell lung cancer: a Canadian national consensus statement. Curr Oncol 16: 27–48. 19229369 10.3747/co.v16i1.393 PMC2644627

[16] Cripps C, Gill S, Ahmed S, Colwell B, Dowden S, et al (2010) Consensus recommendations for the use of anti-egfr therapies in metastatic colorectal cancer. Curr Oncol 17: 39–45. 10.3747/co.v17i6.670 PMC2993438 21151408

[17] Vickers M, Samson B, Colwell B, Cripps C, Jalink D, et al (2010) Eastern Canadian Colorectal Cancer Consensus Conference: setting the limits of resectable disease. Curr Oncol 17: 70–77. 10.3747/co.v17i3.610 PMC2880907 20651901

[18] Kocha W, Maroun J, Kennecke H, Law C, Metrakos P, et al (2010) Consensus recommendations for the diagnosis and management of well-differentiated gastroenterohepatic neuroendocrine tumours: a revised statement from a Canadian National Expert Group. Curr Oncol 17: 49–64. 20567626 10.3747/co.v17i3.484 PMC2880904

[19] Asmis T, Balaa F, Scully L, Papadatos D, Marginean C, et al (2010) Diagnosis and management of hepatocellular carcinoma: results of a consensus meeting of The Ottawa Hospital Cancer Centre. Curr Oncol 17: 6–12. 10.3747/co.v17i2.555 PMC2854641 20404972

[20] Sherman M, Burak K, Maroun J, Metrakos P, Knox JJ, et al (2011) Multidisciplinary Canadian consensus recommendations for the management and treatment of hepatocellular carcinoma. Curr Oncol 18: 228–240. 21980250 10.3747/co.v18i5.952 PMC3185900

[21] Aubin F, Gill S, Burkes R, Colwell B, Kamel-Reid S, et al (2011) Canadian Expert Group consensus recommendations: KRAS testing in colorectal cancer. Curr Oncol 18: e180–184. 21874108 10.3747/co.v18i4.779 PMC3149550

[22] Vickers MM, Pasieka J, Dixon E, McEwan S, McKay A, et al (2012) Report from the 13th annual Western canadian gastrointestinal cancer consensus conference; calgary, alberta; september 8–10, 2011. Curr Oncol 19: e468–477. 23300370 10.3747/co.19.1167 PMC3503677

[23] McEwen S, Egan M, Chasen M, Fitch M (2013) Consensus recommendations for cancer rehabilitation: research and education priorities. Curr Oncol 20: 64–69.

[24] Pritchard KI, Gelmon KA, Rayson D, Provencher L, Webster M, et al (2013) Endocrine therapy for postmenopausal women with hormone receptor-positive her2-negative advanced breast cancer after progression or recurrence on nonsteroidal aromatase inhibitor therapy: a Canadian consensus statement. Curr Oncol 20: 48–61. 23443928 10.3747/co.20.1316 PMC3557331

[25] Di Valentin T, Biagi J, Bourque S, Butt R, Champion P, et al (2013) Eastern Canadian Colorectal Cancer Consensus Conference: standards of care for the treatment of patients with rectal, pancreatic, and gastrointestinal stromal tumours and pancreatic neuroendocrine tumours. Curr Oncol 20: e455–464. 24155642 10.3747/co.20.1638 PMC3805414

[26] Reece D, Imrie K, Stevens A, Smith CA (2006) Bortezomib in multiple myeloma and lymphoma: a systematic review and clinical practice guideline. Curr Oncol 13: 160–172. 22792013 10.3747/co.v13i5.106 PMC3394599

[27] Maroun J, Kocha W, Kvols L, Bjarnason G, Chen E, et al (2006) Guidelines for the diagnosis and management of carcinoid tumours. Part 1: the gastrointestinal tract. A statement from a Canadian National Carcinoid Expert Group. Curr Oncol 13: 67–76. 17576444 10.3390/curroncol13020006 PMC1891174

[28] Jonker D, Rumble RB, Maroun J (2006) Role of oxaliplatin combined with 5-fluorouracil and folinic acid in the first- and second-line treatment of advanced colorectal cancer. Curr Oncol 13: 173–184. 22792014 10.3747/co.v13i5.99 PMC3394600

[29] Mason WP, Maestro RD, Eisenstat D, Forsyth P, Fulton D, et al (2007) Canadian recommendations for the treatment of glioblastoma multiforme. Curr Oncol 14: 110–117. 17593983 10.3747/co.2007.119 PMC1899357

[30] Quirt I, Verma S, Petrella T, Bak K, Charette M, et al. (2007) Temozolomide for the Treatment of Metastatic Melanoma: A Practice Guideline. Current Oncology, {S1}14. 10.3747/co.2007.98 PMC1891190 17576461

[31] Verma S, Younus J, Stys-Norman D, Haynes AE, Blackstein M (2007) Ifosfamide-based combination chemotherapy in advanced soft-tissue sarcoma: a practice guideline. Curr Oncol 14: 144–148. 17710206 10.3747/co.2007.130 PMC1948866

[32] Mintz A, Perry J, Spithoff K, Chambers A, Laperriere N (2007) Management of single brain metastasis: a practice guideline. Curr Oncol 14: 131–143. 17710205 10.3747/co.2007.129 PMC1948870

[33] Fraser G SC, Imrie K, Meyer R (2007) Alemtuzumab in Chronic Lymphocytic Leukemia: A Systematic Review and Clinical Practice Guideline. Current Oncology, {S1}14. 10.3747/co.2007.118 PMC1899355 17593982

[34] Petrella T, Quirt I, Verma S, Haynes AE, Charette M, et al (2007) Single-Agent Interleukin-2 in the Treatment of Metastatic Melanoma: A Clinical Practice Guideline. Curr Oncol 14: 21–26. 17576460 10.3747/co.2007.97 PMC1891192

[35] Verma S, Petrella T, Hamm C, Bak K, Charette M (2008) Biochemotherapy for the treatment of metastatic malignant melanoma: a clinical practice guideline. Curr Oncol 15: 85–89. 18454183 PMC2365480

[36] Verma S, Younus J, Haynes AE, Stys-Norman D, Blackstein M (2008) Dose-intensive chemotherapy with growth factor or autologous bone marrow or stem-cell transplant support in first-line treatment of advanced or metastatic adult soft tissue sarcoma: a clinical practice guideline. Curr Oncol 15: 80–84. 18454188 10.3747/co.v15i2.162 PMC2365487

[37] Cripps C, Winquist E, Devries MC, Stys-Norman D, Gilbert R (2010) Epidermal growth factor receptor targeted therapy in stages III and IV head and neck cancer. Curr Oncol 17: 37–48. 20567625 10.3747/co.v17i3.520 PMC2880902

[38] Elit L, Fyles AW, Oliver TK, Devries-Aboud MC, Fung-Kee-Fung M (2010) Follow-up for women after treatment for cervical cancer. Curr Oncol 17: 65–69. 10.3747/co.v17i3.514 PMC2880906 20567627

[39] Dawson AJ, McGowan-Jordan J, Chernos J, Xu J, Lavoie J, et al (2011) Canadian College of Medical Geneticists guidelines for the indications, analysis, and reporting of cancer specimens. Curr Oncol 18: e250–255. 21980257 10.3747/co.v18i5.775 PMC3185907

[40] Mackenzie M, Spithoff K, Jonker D (2011) Systemic therapy for advanced gastric cancer: a clinical practice guideline. Curr Oncol 18: e202–209. 21874111 10.3747/co.v18i4.737 PMC3149553

[41] Howell D, Hack TF, Oliver TK, Chulak T, Mayo S, et al (2011) Survivorship services for adult cancer populations: a pan-Canadian guideline. Curr Oncol 18: e265–281. 22184494 10.3747/co.v18i6.956 PMC3224035

[42] Darling GE, Dickie AJ, Malthaner RA, Kennedy EB, Tey R (2011) Invasive mediastinal staging of non-small-cell lung cancer: a clinical practice guideline. Curr Oncol 18: e304–310. 22184498 10.3747/co.v18i6.820 PMC3224039

[43] Dodge JE, Covens AL, Lacchetti C, Elit LM, Le T, et al (2012) Management of a suspicious adnexal mass: a clinical practice guideline. Curr Oncol 19: e244–257. 22876153 10.3747/co.19.980 PMC3410836

[44] Chen C, Baldassarre F, Kanjeekal S, Herst J, Hicks L, et al (2013) Lenalidomide in multiple myeloma-a practice guideline. Curr Oncol 20: e136–149. 23559881 10.3747/co.20.1252 PMC3615865

[45] Gupta AA, Yao X, Verma S, Mackay H, Hopkins L (2013) Chemotherapy (gemcitabine, docetaxel plus gemcitabine, doxorubicin, or trabectedin) in inoperable, locally advanced, recurrent, or metastatic uterine leiomyosarcoma: a clinical practice guideline. Curr Oncol 20: e448–454. 24155641 10.3747/co.20.1357 PMC3805413

[46] Yoo J, Lacchetti C, Hammond JA, Gilbert RW (2013) Role of endolaryngeal surgery (with or without laser) compared with radiotherapy in the management of early (T1) glottic cancer: a clinical practice guideline. Curr Oncol 20: e132–135. 23559880 10.3747/co.20.1237 PMC3615864

[47] Kandel R, Coakley N, Werier J, Engel J, Ghert M, et al (2013) Surgical margins and handling of soft-tissue sarcoma in extremities: a clinical practice guideline. Curr Oncol 20: e247–254. 23737694 10.3747/co.20.1308 PMC3671031

[48] Gallinger S, Biagi JJ, Fletcher GG, Nhan C, Ruo L, et al (2013) Liver resection for colorectal cancer metastases. Curr Oncol 20: e255–265. 23737695 10.3747/co.20.1341 PMC3671032

[49] Howell D, Keller-Olaman S, Oliver TK, Hack TF, Broadfield L, et al (2013) A pan-Canadian practice guideline and algorithm: screening, assessment, and supportive care of adults with cancer-related fatigue. Curr Oncol 20: e233–246. 23737693 10.3747/co.20.1302 PMC3671030

[50] Trautinger F, Knobler R, Willemze R, Peris K, Stadler R, et al (2006) EORTC consensus recommendations for the treatment of mycosis fungoides/Sézary syndrome. European journal of cancer (Oxford, England: 1990) 42: 1014–1030. 10.1016/j.ejca.2006.01.025 16574401

[51] Van Cutsem E, Nordlinger B, Adam R, Köhne C-H, Pozzo C, et al (2006) Towards a pan-European consensus on the treatment of patients with colorectal liver metastases. European journal of cancer (Oxford, England: 1990) 42: 2212–2221. 10.1016/j.ejca.2006.04.012 16904315

[52] Zinzani PL, d’Amore F, Bombardieri E, Brammer C, Codina JG, et al (2008) Consensus conference: Implementing treatment recommendations on yttrium-90 immunotherapy in clinical practice – Report of a European workshop. European journal of cancer (Oxford, England: 1990) 44: 366–373. 10.1016/j.ejca.2007.12.008 18194857

[53] Garbe C, Peris K, Hauschild A, Saiag P, Middleton M, et al (2010) Diagnosis and treatment of melanoma: European consensus-based interdisciplinary guideline. European journal of cancer (Oxford, England: 1990) 46: 270–283. 10.1016/j.ejca.2009.10.032 19959353

[54] Amant F, Deckers S, Van Calsteren K, Loibl S, Halaska M, et al (2010) Breast cancer in pregnancy: Recommendations of an international consensus meeting. European journal of cancer (Oxford, England: 1990) 46: 3158–3168. 10.1016/j.ejca.2010.09.010 20932740

[55] Gridelli C, Rossi A, Maione P (2011) 2010 Consensus on Lung Cancer, new clinical recommendations and current status of biomarker assessment – First-line therapy. European journal of cancer (Oxford, England : 1990) 47: S248–S257. 10.1016/S0959-8049(11)70171-X 21943982

[56] Lutz MP, Zalcberg JR, Ducreux M, Ajani JA, Allum W, et al (2012) Highlights of the EORTC St. Gallen International Expert Consensus on the primary therapy of gastric, gastroesophageal and oesophageal cancer – Differential treatment strategies for subtypes of early gastroesophageal cancer. European journal of cancer (Oxford, England: 1990) 48: 2941–2953. 10.1016/j.ejca.2012.07.029 22921186

[57] Garbe C, Peris K, Hauschild A, Saiag P, Middleton M, et al (2012) Diagnosis and treatment of melanoma. European consensus-based interdisciplinary guideline – Update 2012. European journal of cancer (Oxford, England: 1990) 48: 2375–2390. 10.1016/j.ejca.2012.06.013 22981501

[58] German Austrian (2103) Swiss consensus conference on the diagnosis and local treatment of the axilla in breast cancer (2103) European journal of cancer (Oxford, England: 1990). 49: 2277–2283. 10.1016/j.ejca.2013.01.034 23490652

[59] Czauderna P, Otte JB, Aronson DC, Gauthier F, Mackinlay G, et al (2005) Guidelines for surgical treatment of hepatoblastoma in the modern era–recommendations from the Childhood Liver Tumour Strategy Group of the International Society of Paediatric Oncology (SIOPEL). Eur J Cancer 41: 1031–1036. 15862752 10.1016/j.ejca.2005.02.004

[60] Becker G, Galandi D, Blum HE (2006) Malignant ascites: systematic review and guideline for treatment. Eur J Cancer 42: 589–597. 16434188 10.1016/j.ejca.2005.11.018

[61] Aapro MS, Cameron DA, Pettengell R, Bohlius J, Crawford J, et al (2006) EORTC guidelines for the use of granulocyte-colony stimulating factor to reduce the incidence of chemotherapy-induced febrile neutropenia in adult patients with lymphomas and solid tumours. Eur J Cancer 42: 2433–2453. 16750358 10.1016/j.ejca.2006.05.002

[62] Bokemeyer C, Aapro MS, Courdi A, Foubert J, Link H, et al (2007) EORTC guidelines for the use of erythropoietic proteins in anaemic patients with cancer: 2006 update. Eur J Cancer 43: 258–270. 17182241 10.1016/j.ejca.2006.10.014

[63] Duffy MJ, van Dalen A, Haglund C, Hansson L, Holinski-Feder E, et al (2007) Tumour markers in colorectal cancer: European Group on Tumour Markers (EGTM) guidelines for clinical use. Eur J Cancer 43: 1348–1360. 17512720 10.1016/j.ejca.2007.03.021

[64] Cataliotti L, De Wolf C, Holland R, Marotti L, Perry N, et al (2007) Guidelines on the standards for the training of specialised health professionals dealing with breast cancer. Eur J Cancer 43: 660–675. 17276672 10.1016/j.ejca.2006.12.008

[65] Quinn B, Potting CM, Stone R, Blijlevens NM, Fliedner M, et al (2008) Guidelines for the assessment of oral mucositis in adult chemotherapy, radiotherapy and haematopoietic stem cell transplant patients. Eur J Cancer 44: 61–72. 17988858 10.1016/j.ejca.2007.09.014

[66] Maschmeyer G, Beinert T, Buchheidt D, Cornely OA, Einsele H, et al (2009) Diagnosis and antimicrobial therapy of lung infiltrates in febrile neutropenic patients: Guidelines of the infectious diseases working party of the German Society of Haematology and Oncology. Eur J Cancer 45: 2462–2472. 19467584 10.1016/j.ejca.2009.05.001

[67] Glenny AM, Gibson F, Auld E, Coulson S, Clarkson JE, et al. The development of evidence-based guidelines on mouth care for children, teenagers and young adults treated for cancer. European Journal of Cancer 46: 1399–1412. 10.1016/j.ejca.2010.01.023 20227272

[68] Aapro MS, Bohlius J, Cameron DA, Dal Lago L, Donnelly JP, et al (2011) 2010 update of EORTC guidelines for the use of granulocyte-colony stimulating factor to reduce the incidence of chemotherapy-induced febrile neutropenia in adult patients with lymphoproliferative disorders and solid tumours. Eur J Cancer 47: 8–32. 21095116 10.1016/j.ejca.2010.10.013

[69] Rayner L, Price A, Hotopf M, Higginson IJ (2011) The development of evidence-based European guidelines on the management of depression in palliative cancer care. Eur J Cancer 47: 702–712. 21211961 10.1016/j.ejca.2010.11.027

[70] Herkert JC, Niessen RC, Olderode-Berends MJ, Veenstra-Knol HE, Vos YJ, et al (2011) Paediatric intestinal cancer and polyposis due to bi-allelic PMS2 mutations: case series, review and follow-up guidelines. Eur J Cancer 47: 965–982. 21376568 10.1016/j.ejca.2011.01.013

[71] EASL-EORTC clinical practice guidelines: management of hepatocellular carcinoma. Eur J Cancer 48: 599–641. 22424278 10.1016/j.ejca.2011.12.021

[72] Juweid ME, Stroobants S, Hoekstra OS, Mottaghy FM, Dietlein M, et al (2007) Use of Positron Emission Tomography for Response Assessment of Lymphoma: Consensus of the Imaging Subcommittee of International Harmonization Project in Lymphoma. Journal of Clinical Oncology 25: 571–578. 17242397 10.1200/JCO.2006.08.2305

[73] Ponzoni M, Ferreri AJM, Campo E, Facchetti F, Mazzucchelli L, et al (2007) Definition, Diagnosis, and Management of Intravascular Large B-Cell Lymphoma: Proposals and Perspectives From an International Consensus Meeting. Journal of Clinical Oncology 25: 3168–3173. 17577023 10.1200/JCO.2006.08.2313

[74] Philip PA, Mooney M, Jaffe D, Eckhardt G, Moore M, et al (2009) Consensus Report of the National Cancer Institute Clinical Trials Planning Meeting on Pancreas Cancer Treatment. Journal of Clinical Oncology 27: 5660–5669. 19858397 10.1200/JCO.2009.21.9022 PMC7587401

[75] Tsukasaki K, Hermine O, Bazarbachi A, Ratner L, Ramos JC, et al (2009) Definition, Prognostic Factors, Treatment, and Response Criteria of Adult T-Cell Leukemia-Lymphoma: A Proposal From an International Consensus Meeting. Journal of Clinical Oncology 27: 453–459. 19064971 10.1200/JCO.2008.18.2428 PMC2737379

[76] Lokhorst H, Einsele H, Vesole D, Bruno B, Miguel JS, et al (2010) International Myeloma Working Group Consensus Statement Regarding the Current Status of Allogeneic Stem-Cell Transplantation for Multiple Myeloma. Journal of Clinical Oncology 28: 4521–4530. 20697091 10.1200/JCO.2010.29.7929

[77] Dimopoulos MA, Terpos E, Chanan-Khan A, Leung N, Ludwig H, et al (2010) Renal Impairment in Patients With Multiple Myeloma: A Consensus Statement on Behalf of the International Myeloma Working Group. Journal of Clinical Oncology 28: 4976–4984. 20956629 10.1200/JCO.2010.30.8791

[78] Thomas MB, Jaffe D, Choti MM, Belghiti J, Curley S, et al (2010) Hepatocellular Carcinoma: Consensus Recommendations of the National Cancer Institute Clinical Trials Planning Meeting. Journal of Clinical Oncology 28: 3994–4005. 20679622 10.1200/JCO.2010.28.7805 PMC2940397

[79] Kulke MH, Siu LL, Tepper JE, Fisher G, Jaffe D, et al (2011) Future Directions in the Treatment of Neuroendocrine Tumors: Consensus Report of the National Cancer Institute Neuroendocrine Tumor Clinical Trials Planning Meeting. Journal of Clinical Oncology 29: 934–943. 21263089 10.1200/JCO.2010.33.2056 PMC3068065

[80] Olsen EA, Whittaker S, Kim YH, Duvic M, Prince HM, et al (2011) Clinical End Points and Response Criteria in Mycosis Fungoides and Sézary Syndrome: A Consensus Statement of the International Society for Cutaneous Lymphomas, the United States Cutaneous Lymphoma Consortium, and the Cutaneous Lymphoma Task Force of the European Organisation for Research and Treatment of Cancer. Journal of Clinical Oncology 29: 2598–2607. 21576639 10.1200/JCO.2010.32.0630 PMC3422534

[81] Brock PR, Knight KR, Freyer DR, Campbell KCM, Steyger PS, et al (2012) Platinum-Induced Ototoxicity in Children: A Consensus Review on Mechanisms, Predisposition, and Protection, Including a New International Society of Pediatric Oncology Boston Ototoxicity Scale. Journal of Clinical Oncology 30: 2408–2417. 22547603 10.1200/JCO.2011.39.1110 PMC3675696

[82] Lyman GH, Giuliano AE, Somerfield MR, Benson AB 3rd, Bodurka DC, et al (2005) American Society of Clinical Oncology guideline recommendations for sentinel lymph node biopsy in early-stage breast cancer. J Clin Oncol 23: 7703–7720. 16157938 10.1200/JCO.2005.08.001

[83] Desch CE, Benson AB, Somerfield MR, Flynn PJ, Krause C, et al (2005) Colorectal Cancer Surveillance: 2005 Update of an American Society of Clinical Oncology Practice Guideline. Journal of Clinical Oncology 23: 8512–8519. 16260687 10.1200/JCO.2005.04.0063

[84] Wolff AC, Hammond MEH, Schwartz JN, Hagerty KL, Allred DC, et al (2006) American Society of Clinical Oncology/College of American Pathologists Guideline Recommendations for Human Epidermal Growth Factor Receptor 2 Testing in Breast Cancer. Journal of Clinical Oncology 25: 118–145. 17159189 10.1200/JCO.2006.09.2775

[85] Smith TJ, Khatcheressian J, Lyman GH, Ozer H, Armitage JO, et al (2006) 2006 Update of Recommendations for the Use of White Blood Cell Growth Factors: An Evidence-Based Clinical Practice Guideline. Journal of Clinical Oncology 24: 3187–3205. 16682719 10.1200/JCO.2006.06.4451

[86] Pfister DG, Laurie SA, Weinstein GS, Mendenhall WM, Adelstein DJ, et al (2006) American Society of Clinical Oncology Clinical Practice Guideline for the Use of Larynx-Preservation Strategies in the Treatment of Laryngeal Cancer. Journal of Clinical Oncology 24: 3693–3704. 16832122 10.1200/JCO.2006.07.4559

[87] Kris MG, Hesketh PJ, Somerfield MR, Feyer P, Clark-Snow R, et al (2006) American Society of Clinical Oncology Guideline for Antiemetics in Oncology: Update 2006. Journal of Clinical Oncology 24: 2932–2947. 16717289 10.1200/JCO.2006.06.9591

[88] Lyman GH, Khorana AA, Falanga A, Clarke-Pearson D, Flowers C, et al (2007) American Society of Clinical Oncology Guideline: Recommendations for Venous Thromboembolism Prophylaxis and Treatment in Patients With Cancer. Journal of Clinical Oncology 25: 5490–5505. 17968019 10.1200/JCO.2007.14.1283

[89] Pisters KMW, Evans WK, Azzoli CG, Kris MG, Smith CA, et al (2007) Cancer Care Ontario and American Society of Clinical Oncology Adjuvant Chemotherapy and Adjuvant Radiation Therapy for Stages I-IIIA Resectable Non–Small-Cell Lung Cancer Guideline. Journal of Clinical Oncology 25: 5506–5518. 17954710 10.1200/JCO.2007.14.1226

[90] Basch EM, Somerfield MR, Beer TM, Carducci MA, Higano CS, et al (2007) American Society of Clinical Oncology Endorsement of the Cancer Care Ontario Practice Guideline on Nonhormonal Therapy for Men With Metastatic Hormone-Refractory (castration-resistant) Prostate Cancer. Journal of Clinical Oncology 25: 5313–5318. 17925542 10.1200/JCO.2007.13.4536

[91] Kyle RA, Yee GC, Somerfield MR, Flynn PJ, Halabi S, et al (2007) American Society of Clinical Oncology 2007 Clinical Practice Guideline Update on the Role of Bisphosphonates in Multiple Myeloma. Journal of Clinical Oncology 25: 2464–2472. 17515569 10.1200/JCO.2007.12.1269

[92] Loblaw DA, Virgo KS, Nam R, Somerfield MR, Ben-Josef E, et al (2007) Initial Hormonal Management of Androgen-Sensitive Metastatic, Recurrent, or Progressive Prostate Cancer: 2007 Update of an American Society of Clinical Oncology Practice Guideline. Journal of Clinical Oncology 25: 1596–1605. 17404365 10.1200/JCO.2006.10.1949

[93] Hensley ML, Hagerty KL, Kewalramani T, Green DM, Meropol NJ, et al (2009) American Society of Clinical Oncology 2008 Clinical Practice Guideline Update: Use of Chemotherapy and Radiation Therapy Protectants. Journal of Clinical Oncology 27: 127–145. 19018081 10.1200/JCO.2008.17.2627

[94] Kramer BS, Hagerty KL, Justman S, Somerfield MR, Albertsen PC, et al (2009) Use of 5-alpha-reductase inhibitors for prostate cancer chemoprevention: American Society of Clinical Oncology/American Urological Association 2008 Clinical Practice Guideline. J Clin Oncol 27: 1502–1516. 19252137 10.1200/JCO.2008.16.9599 PMC2668556

[95] Azzoli CG, Baker S, Temin S, Pao W, Aliff T, et al (2009) American Society of Clinical Oncology Clinical Practice Guideline Update on Chemotherapy for Stage IV Non–Small-Cell Lung Cancer. Journal of Clinical Oncology 27: 6251–6266. 19917871 10.1200/JCO.2009.23.5622 PMC2793036

[96] Visvanathan K, Chlebowski RT, Hurley P, Col NF, Ropka M, et al (2009) American Society of Clinical Oncology Clinical Practice Guideline Update on the Use of Pharmacologic Interventions Including Tamoxifen, Raloxifene, and Aromatase Inhibition for Breast Cancer Risk Reduction. Journal of Clinical Oncology 27: 3235–3258. 19470930 10.1200/JCO.2008.20.5179 PMC2716943

[97] Rizzo JD, Brouwers M, Hurley P, Seidenfeld J, Arcasoy MO, et al (2010) American Society of Clinical Oncology/American Society of Hematology Clinical Practice Guideline Update on the Use of Epoetin and Darbepoetin in Adult Patients With Cancer. Journal of Clinical Oncology 28: 4996–5010. 20975064 10.1200/JCO.2010.29.2201

[98] Burstein HJ, Prestrud AA, Seidenfeld J, Anderson H, Buchholz TA, et al (2010) American Society of Clinical Oncology Clinical Practice Guideline: Update on Adjuvant Endocrine Therapy for Women With Hormone Receptor–Positive Breast Cancer. Journal of Clinical Oncology 28: 3784–3796. 20625130 10.1200/JCO.2009.26.3756 PMC5569672

[99] Gilligan TD, Seidenfeld J, Basch EM, Einhorn LH, Fancher T, et al (2010) American Society of Clinical Oncology Clinical Practice Guideline on Uses of Serum Tumor Markers in Adult Males With Germ Cell Tumors. Journal of Clinical Oncology 28: 3388–3404. 20530278 10.1200/JCO.2009.26.4481

[100] Hammond MEH, Hayes DF, Dowsett M, Allred DC, Hagerty KL, et al (2010) American Society of Clinical Oncology/College of American Pathologists Guideline Recommendations for Immunohistochemical Testing of Estrogen and Progesterone Receptors in Breast Cancer. Journal of Clinical Oncology 28: 2784–2795. 20404251 10.1200/JCO.2009.25.6529 PMC2881855

[101] Basch E, Prestrud AA, Hesketh PJ, Kris MG, Feyer PC, et al (2011) Antiemetics: American Society of Clinical Oncology Clinical Practice Guideline Update. Journal of Clinical Oncology 29: 4189–4198. 21947834 10.1200/JCO.2010.34.4614 PMC4876353

[102] Griggs JJ, Somerfield MR, Anderson H, Henry NL, Hudis CA, et al (2011) American Society of Clinical Oncology Endorsement of the Cancer Care Ontario Practice Guideline on Adjuvant Ovarian Ablation in the Treatment of Premenopausal Women With Early-Stage Invasive Breast Cancer. Journal of Clinical Oncology 29: 3939–3942. 21900112 10.1200/JCO.2011.36.4950

[103] Azzoli CG, Temin S, Aliff T, Baker S, Brahmer J, et al (2011) 2011 Focused Update of 2009 American Society of Clinical Oncology Clinical Practice Guideline Update on Chemotherapy for Stage IV Non–Small-Cell Lung Cancer. Journal of Clinical Oncology 29: 3825–3831. 21900105 10.1200/JCO.2010.34.2774 PMC3675703

[104] Burstein HJ, Mangu PB, Somerfield MR, Schrag D, Samson D, et al (2011) American Society of Clinical Oncology Clinical Practice Guideline Update on the Use of Chemotherapy Sensitivity and Resistance Assays. Journal of Clinical Oncology 29: 3328–3330. 21788567 10.1200/JCO.2011.36.0354

[105] Wong SL, Balch CM, Hurley P, Agarwala SS, Akhurst TJ, et al (2012) Sentinel Lymph Node Biopsy for Melanoma: American Society of Clinical Oncology and Society of Surgical Oncology Joint Clinical Practice Guideline. Journal of Clinical Oncology 30: 2912–2918. 22778321 10.1200/JCO.2011.40.3519 PMC5950498

[106] Griggs JJ, Mangu PB, Anderson H, Balaban EP, Dignam JJ, et al (2012) Appropriate Chemotherapy Dosing for Obese Adult Patients With Cancer: American Society of Clinical Oncology Clinical Practice Guideline. Journal of Clinical Oncology 30: 1553–1561. 22473167 10.1200/JCO.2011.39.9436

[107] Visvanathan K, Hurley P, Bantug E, Brown P, Col NF, et al (2013) Use of Pharmacologic Interventions for Breast Cancer Risk Reduction: American Society of Clinical Oncology Clinical Practice Guideline. Journal of Clinical Oncology 31: 2942–2962. 23835710 10.1200/JCO.2013.49.3122

[108] Loren AW, Mangu PB, Beck LN, Brennan L, Magdalinski AJ, et al (2013) Fertility Preservation for Patients With Cancer: American Society of Clinical Oncology Clinical Practice Guideline Update. Journal of Clinical Oncology 31: 2500–2510. 23715580 10.1200/JCO.2013.49.2678 PMC5321083

[109] Schiffer CA, Mangu PB, Wade JC, Camp-Sorrell D, Cope DG, et al (2013) Central venous catheter care for the patient with cancer: American Society of Clinical Oncology clinical practice guideline. J Clin Oncol 31: 1357–1370. 23460705 10.1200/JCO.2012.45.5733

[110] Khatcheressian JL, Hurley P, Bantug E, Esserman LJ, Grunfeld E, et al (2013) Breast Cancer Follow-Up and Management After Primary Treatment: American Society of Clinical Oncology Clinical Practice Guideline Update. Journal of Clinical Oncology 31: 961–965. 23129741 10.1200/JCO.2012.45.9859

[111] Flowers CR, Seidenfeld J, Bow EJ, Karten C, Gleason C, et al (2013) Antimicrobial Prophylaxis and Outpatient Management of Fever and Neutropenia in Adults Treated for Malignancy: American Society of Clinical Oncology Clinical Practice Guideline. Journal of Clinical Oncology 31: 794–810. 23319691 10.1200/JCO.2012.45.8661

[112] Fervers B, Burgers JS, Haugh MC, Brouwers M, Browman G, et al (2005) Predictors of high quality clinical practice guidelines: examples in oncology. Int J Qual Health Care 17: 123–132. 15665068 10.1093/intqhc/mzi011

[113] Burgers JS, Fervers B, Haugh M, Brouwers M, Browman G, et al (2004) International assessment of the quality of clinical practice guidelines in oncology using the Appraisal of Guidelines and Research and Evaluation Instrument. J Clin Oncol 22: 2000–2007. 15143093 10.1200/JCO.2004.06.157

[114] de Haas ER, de Vijlder HC, van Reesema WS, van Everdingen JJ, Neumann HA (2007) Quality of clinical practice guidelines in dermatological oncology. J Eur Acad Dermatol Venereol 21: 1193–1198. 17894704 10.1111/j.1468-3083.2007.02216.x

[115] Shimbo T, Fukui T, Ishioka C, Okamoto K, Okamoto T, et al (2010) Quality of guideline development assessed by the Evaluation Committee of the Japan Society of Clinical Oncology. Int J Clin Oncol 15: 227–233. 20333432 10.1007/s10147-010-0060-y

[116] Langton JM, Drew AK, Mellish L, Olivier J, Ward RL, et al (2011) The quality of web-based oncology guidelines and protocols: how do international sites stack up? Br J Cancer 105: 1166–1172. 21934686 10.1038/bjc.2011.378 PMC3208501

[117] Hogeveen SE, Han D, Trudeau-Tavara S, Buck J, Brezden-Masley CB, et al (2012) Comparison of international breast cancer guidelines: are we globally consistent? cancer guideline AGREEment. Curr Oncol 19: e184–190. 22670108 10.3747/co.19.930 PMC3364779

[118] Lopez-Olivo MA, Kallen MA, Ortiz Z, Skidmore B, Suarez-Almazor ME (2008) Quality appraisal of clinical practice guidelines and consensus statements on the use of biologic agents in rheumatoid arthritis: a systematic review. Arthritis Rheum 59: 1625–1638. 18975351 10.1002/art.24207

[119] Collaboration TC (2014) Understanding Searching Techniques to Inform HTA, Systematic Reviews and Guideline Development.

[120] Graham R, Mancher M, Wolman DM, Greenfield S, Steinberg E (2011) Clinical Practice Guidelines We Can Trust. The National Academies Press. 24983061

[121] Mendelson TB, Meltzer M, Campbell EG, Caplan AL, Kirkpatrick JN (2011) COnflicts of interest in cardiovascular clinical practice guidelines. Archives of Internal Medicine 171: 577–584. 21444849 10.1001/archinternmed.2011.96

[122] American Society of Clinical Oncology (2013) Conflict of Interest Policy Implementation for Clinical Practice Guidelines of American Society of Clinical Oncology. http://www.asco.org/sites/www.asco.org/files/conflict_of_interest_policy_implementation_for_clinical_practice_guidelines_8.8.2013_0.pdf [Accessed 28 May 2014].

[123] Norris SL, Holmer HK, Ogden LA, Burda BU (2011) Conflict of interest in clinical practice guideline development: a systematic review. PLoS One 6: e25153. 22039406 10.1371/journal.pone.0025153 PMC3198464

[124] Cosgrove L, Bursztajn HJ, Erlich DR, Wheeler EE, Shaughnessy AF (2013) Conflicts of interest and the quality of recommendations in clinical guidelines. Journal of Evaluation in Clinical Practice 19: 674–681. 23731207 10.1111/jep.12016

