# Correction: Clinical Practice Guidelines and Consensus Statements in Oncology – An Assessment of Their Methodological Quality

**DOI:** 10.1371/journal.pone.0116267

**Published:** 2014-12-17

**Authors:** The PLOS ONE Staff

Figures 1 and 2 are incorrect. Please view the corrected Figures 1 and 2 here.

**Figure 1:**
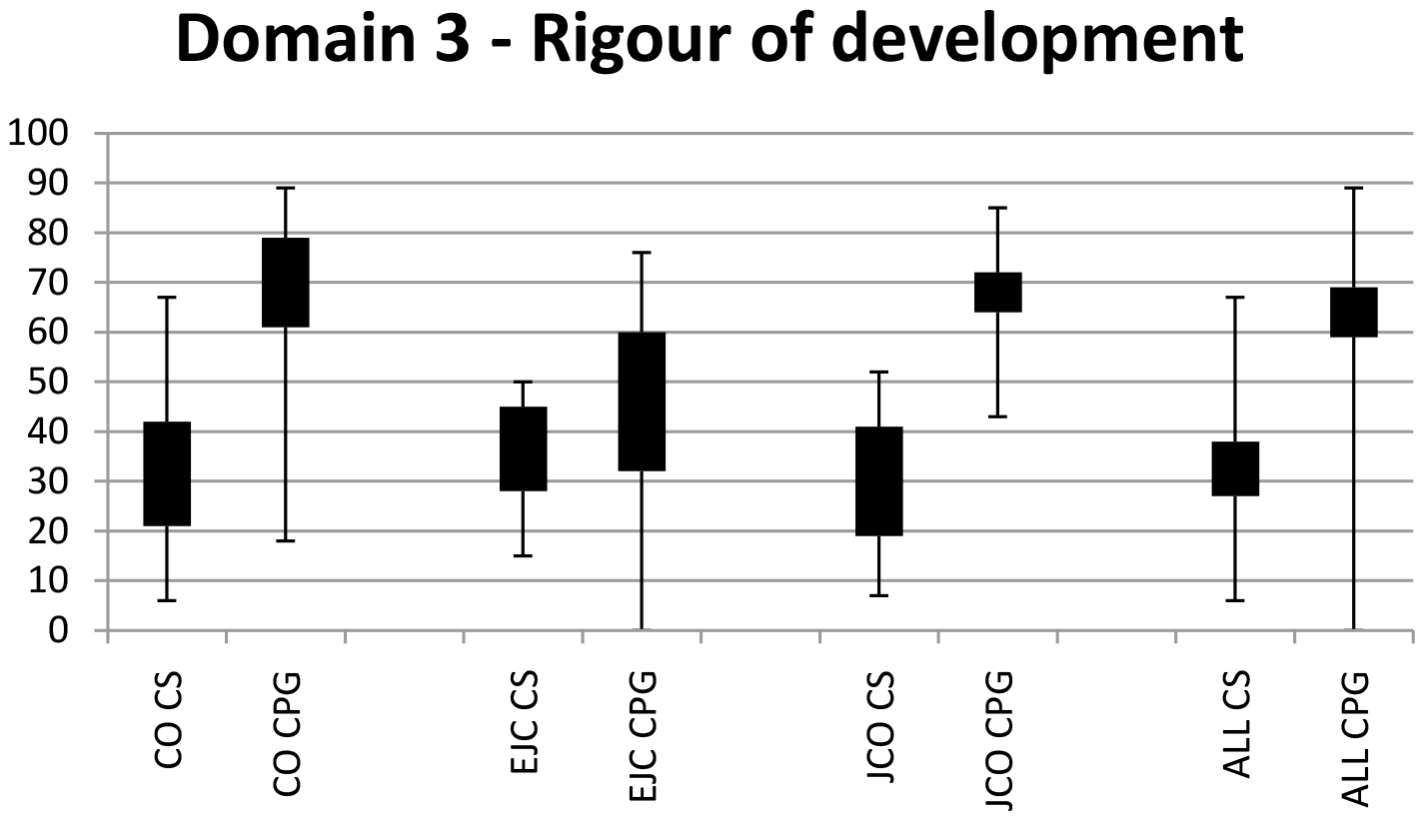
Range and 95% confidence intervals for Rigour of development scores. CO  =  Current Oncology. EJC  =  European Journal of Cancer**.** CS  =  Consensus statements. JCO  =  Journal of Clinical Oncology**.** CPG  =  Clinical practice guidelines.

**Figure 2:**
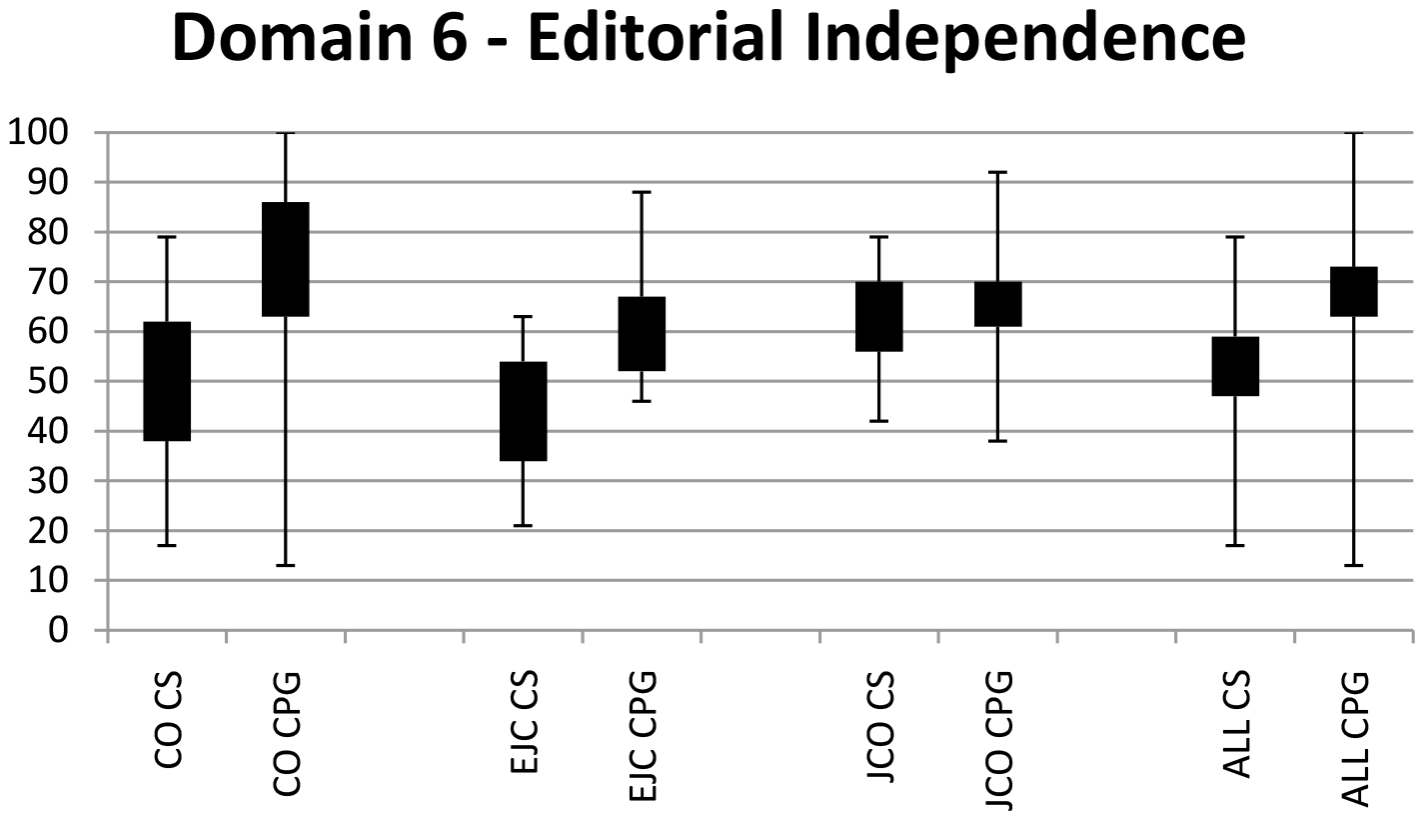
Range and 95% confidence intervals for Editorial independence scores. CO  =  Current Oncology. EJC  =  European Journal of Cancer. CS  =  Consensus statements. JCO  =  Journal of Clinical Oncology. CPG  =  Clinical practice guidelines.
